# CryoEM at 100 keV: a demonstration and prospects

**DOI:** 10.1107/S2052252519012612

**Published:** 2019-10-11

**Authors:** K. Naydenova, G. McMullan, M. J. Peet, Y. Lee, P. C. Edwards, S. Chen, E. Leahy, S. Scotcher, R. Henderson, C. J. Russo

**Affiliations:** a MRC Laboratory of Molecular Biology, Cambridge CB2 0QH, England

**Keywords:** advances in microscope hardware, single-particle cryoEM, electron cryomicroscopy, low-dose electron microscopy, structure determination, single-particle reconstruction, direct detectors

## Abstract

100 kV is investigated as the operating voltage for single-particle electron cryomicroscopy (cryoEM). Reducing the electron energy from the current standard of 300 or 200 keV offers both cost savings and potentially improved imaging. The latter follows from recent measurements of radiation damage to biological specimens by high-energy electrons, which show that at lower energies there is an increased amount of information available per unit damage. For frozen hydrated specimens around 300 Å in thickness, the predicted optimal electron energy for imaging is 100 keV. Currently available electron cryomicroscopes in the 100–120 keV range are not optimized for cryoEM as they lack both the spatially coherent illumination needed for the high defocus used in cryoEM and imaging detectors optimized for 100 keV electrons. To demonstrate the potential of imaging at 100 kV, the voltage of a standard, commercial 200 kV field-emission gun (FEG) microscope was reduced to 100 kV and a side-entry cryoholder was used. As high-efficiency, large-area cameras are not currently available for 100 keV electrons, a commercial hybrid pixel camera designed for X-ray detection was attached to the camera chamber and was used for low-dose data collection. Using this configuration, five single-particle specimens were imaged: hepatitis B virus capsid, bacterial 70S ribosome, catalase, DNA protection during starvation protein and haemoglobin, ranging in size from 4.5 MDa to 64 kDa with corresponding diameters from 320 to 72 Å. These five data sets were used to reconstruct 3D structures with resolutions between 8.4 and 3.4 Å. Based on this work, the practical advantages and current technological limitations to single-particle cryoEM at 100 keV are considered. These results are also discussed in the context of future microscope development towards the goal of rapid, simple and widely available structure determination of any purified biological specimen.

## Abstract 2

Several structures were determined by single-particle cryoEM at 100 keV and the implications for future electron cryomicroscopes are considered.

## 1. Introduction   

In less than ten years, electron cryomicroscopy (cryoEM) has become a mainstream technique in structural biology. Today, high-end 300 keV electron microscopes, equipped with high detective quantum efficiency (DQE) direct electron detectors and powerful computing, are routinely being used to determine relatively easily structures that would have been thought impossible just a few years ago (Kühlbrandt, 2014 ▸; Crowther, 2016 ▸). Still, electron cryomicroscopes are not optimized for structural biology from the point of view of efficient structure determination or cost. Recent work examined the role of electron energy quantitatively for cryoEM of a biological specimen embedded in vitrified water (Peet *et al.*, 2019 ▸). This work showed theory and experiments to determine the optimum electron energy for a biological specimen of a given thickness. The conclusion was that there is a potential improvement of up to 25% in the amount of information available per unit damage for typical single-particle cryoEM specimens, and this could be realized by reducing the energy of the electron beam from the typical 300 or 200 keV to 100 keV. Furthermore, the initial expense and running costs of high-end electron microscopes present a significant barrier to the widespread adoption of cryoEM (Vinothkumar & Henderson, 2016 ▸). A typical high-end instrument has a marginal cost of about £3000 per day to operate (Passmore & Russo, 2016 ▸), corresponding to a full cost of more than £5000 per day, which is well beyond what most individual laboratories that would potentially benefit from the use of cryoEM could afford.

In its simplest form, an electron cryomicroscope must have a bright electron source, a stable stage, a low ice-contamination rate and a large-area, high-efficiency imaging detector. The first three are available in various commercial instruments, but not together in a single microscope designed for high-resolution 100 keV imaging. Optimized large-area imaging detectors for 100 keV electrons are not currently available from any vendor, thus making the detector the limiting technological factor in establishing cryoEM at 100 keV. The same scattering processes that control electron interaction with the specimen also limit scattering within the electron detector (McMullan, Chen *et al.*, 2009 ▸; Faruqi & McMullan, 2018 ▸). A 300 keV electron has a range of 350 µm in silicon (McMullan *et al.*, 2007 ▸), and back-thinning a sensor to 30 µm results in most incident electrons being transmitted through the sensor with very little transverse scattering. A pixel size of ∼8 µm can therefore be used to record the arrival position and hence the spatial information contained in the electron beam. The energy deposited in the sensitive layer of the sensor is sufficient to record almost every electron, and the effect of intrinsic variability in the magnitude of the energy deposited is removed by operating in a counting mode (McMullan, Chen *et al.*, 2009 ▸; McMullan, Clark *et al.*, 2009 ▸; Li *et al.*, 2013 ▸). Monte Carlo simulations of electron trajectories show that 100 keV electrons have a range of 50–60 µm in silicon (McMullan *et al.*, 2007 ▸). This makes it impractical to back-thin a detector to a thickness at which most of the incident electrons would be transmitted, and also sets a lower bound for the optimal pixel size of 60 µm for a silicon sensitive layer. Detectors optimized for 300 keV electrons can be used at 100 keV by binning their output to give ∼60 µm pixels, but they will have a much smaller field of view, reduced dynamic range and unnecessary computational overhead in the data-output stream.

Hybrid pixel detectors offer an alternative for imaging 100 keV electrons (Faruqi & McMullan, 2018 ▸). Originally designed for particle physics (Heijne *et al.*, 1994 ▸), hybrid pixel detectors have recently found wide success in structural biology for photon counting in X-ray detection work (Dinapoli *et al.*, 2011 ▸; Casanas *et al.*, 2016 ▸). In these detectors, incident electrons strike a thick semiconductor layer that is fully depleted via an applied bias voltage. Energy lost by an incident electron generates charge in the layer that is collected by an array of bump bonds (each comprising a pixel) to a specifically designed readout chip (application-specific integrated circuit; ASIC) containing charge-shaping, thresholding and counting electronics. The bump bonds are typically spaced from 55 to 75 µm apart and define the pitch of the pixel array. As the electronics associated with each bump bond work independently, hybrid pixel detectors can operate at very high speeds (∼10 kHz frame rate). Hybrid pixel detectors have the added advantage of being impervious to high exposure to a 100 keV electron beam, and their application in electron microscopy and crystallography has been explored (Faruqi *et al.*, 2005 ▸; Mir *et al.*, 2017 ▸; Tinti *et al.*, 2018 ▸). For example, it is possible to switch to and from diffraction mode with no damage to the detector, and to use the same detector for alignment, search and imaging. For application in cryoEM the main limitation is the difficulty in making suitably large (≥2k × 2k) gapless detectors.

Here, we present a demonstration of cryoEM using 100 keV electrons with the currently available technology assembled together in our laboratory, and then consider the future developments that are needed to make this technology practical and widely available to biologists in general. It is relatively straightforward to change the energy of the electron microscope, and remarkably so when compared with other high-energy particles such as X-rays: it is simply a matter of turning down the voltage on the source and the current in the lenses, with some realignment of the column, which can be performed in a few hours. Recent work has also shown that high-resolution cryoEM is possible at 200 keV (Herzik *et al.*, 2017 ▸, 2019 ▸). For this demonstration, we took a commonly available 200 keV cryomicroscope (FEI TF20), reconfigured the column alignments and optics for 100 keV, attached a hybrid pixel detector (Dectris EIGER; Dinapoli *et al.*, 2011 ▸) to the bottom of the projection chamber and used a standard side-entry cryo-specimen holder (Gatan 626) for imaging cryo-specimens. This configuration was set up in one day and data were collected from several specimens over the course of a week before the microscope was returned to its original configuration.

## 2. Methods and materials   

### 2.1. Detector setup and characterization   

**Figure 1:**
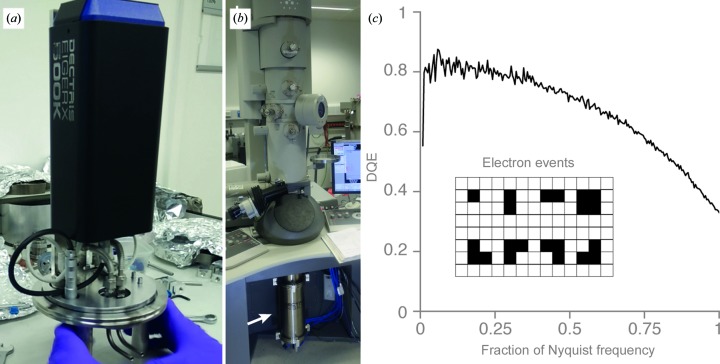
Hybrid pixel detector for data collection at 100 keV. (*a*) shows the EIGER X 500K camera mounted on the ISO160-K flange. (*b*) shows the vacuum enclosure containing the detector fitted to the bottom of the projection chamber of an FEI TF20. (*c*) shows the measured detective quantum efficiency (DQE) of the detector versus spatial frequency (fraction of the Nyquist frequency) at 100 keV. The inset shows the events that are observed with a threshold of 6.5 keV (with probability greater than 0.01%) in images that have less than one incident 100 keV electron per 1000 pixels. The relative probabilities for an electron to be recorded in one, two, three or four pixels are 0.257, 0.520, 0.140 and 0.081, respectively. This distribution for recording incident electrons gives an average response per electron of 2.0 counts and an upper bound for DQE(0) of 0.85.

A hybrid pixel detector (Dectris EIGER X 500K) was used in this study. It has a 75 µm pixel and is composed of eight 256 × 256 pixel modules bump-bonded in a 4 × 2 array to a 450 µm thick silicon sensor. There is a two-pixel spacing between the modules and so the detector size is effectively 1030 × 514 pixels. The EIGER is designed for X-ray detection; to image electrons it was placed in a vacuum housing with an opening for the beam at the top of the chamber. This was made by the MRC LMB workshop and is shown in Fig. 1 ▸(*a*). The detector enclosure was supported within the chamber by an arm mounted to an ISO160-K stainless-steel blanking flange. The flange also contains feedthroughs for the power, data cables and cooling water needed by the detector. The assembly was placed inside a 350 mm ISO160-K straight coupler (Vacuum Services Ltd) and was connected to the existing camera mount below the projection chamber of the microscope by a stainless-steel adaptor [Fig. 1 ▸(*b*)]. All beam-facing surfaces of the adaptor and coupler were lined with 0.5 mm aluminium backed by 4.0 mm of overlapping lead. To reduce counts from stray X-rays, the field of view from the sensor was restricted by replacing the existing beam mask on the camera mount with a rectangular mask matched to the pixel array of the EIGER.

One important parameter to control on a hybrid pixel detector of this type is the event-detection threshold. Here, we set the threshold to 6.5 keV, which was the lowest setting that allowed continuous readout of four-bit data at a 9 kHz frame rate. A low threshold ensured that essentially every electron was detected, but the DQE at zero spatial frequency, DQE(0), was less than 100% as electrons can still be simultaneously recorded in more than one pixel. For 100 keV electrons incident on the 75 µm pixel of the EIGER operating with a 6.5 keV threshold, eight distinct types of single-electron events were recorded [Fig. 1 ▸(*c*), inset]. This limits DQE(0) to at most 85%. In practice, the actual DQE will be slightly lower than the expected value owing to the detection of stray X-rays, cosmic rays and a few uncounted back-scattered electrons. The DQE, as measured by the technique described in McMullan *et al.* (2014 ▸), is shown in Fig. 1 ▸(*c*).

**Figure 2:**
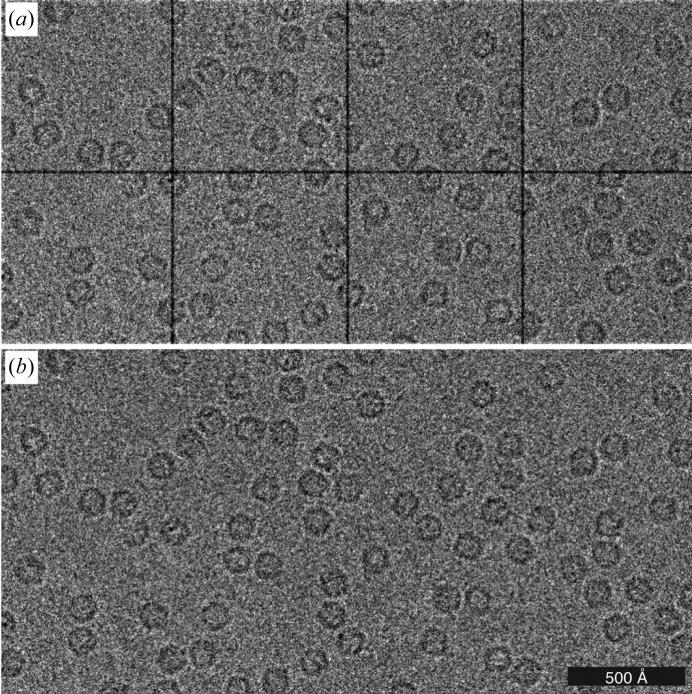
Example micrographs of apoferritin using 100 keV electrons and a hybrid pixel detector (EIGER X 500 K). (*a*) The raw micrograph is separated into eight sectors corresponding to each of the ASIC modules with a two-pixel gap between them. (*b*) The gaps can be removed by gain correction, since the charge between the modules is collected in the neighbouring pixels.

The output from the detector was mirrored to a second capture computer with custom software designed to provide a simple interface for low-dose data collection. Charge generated in the gap between modules is collected in neighbouring pixels, so the application of a suitable gain correction (Fig. 2 ▸) results in a gapless image (1030 × 514 pixels) corresponding to an active detector area of 77.25 × 38.55 mm. The computer also controlled the microscope blanker; depending on the desired mode the data could either be ignored, viewed interactively or stored. For interactive viewing the data stream was binned and displayed at up to 10 frames per second with an optional live power spectrum for use in determining the defocus or other alignments. Data were acquired manually with the existing microscope low-dose software, apart from during exposure mode, in which blanker control was transferred to the data-capture computer. Typically, 2 s exposures were taken; with this arrangement it was possible to acquire approximately one image per minute during manual data collection on a typical cryo-specimen.

### 2.2. DPS cloning and expression   

*Escherichia coli* DNA protection during starvation protein (DPS; Wolf *et al.*, 1999 ▸; amino acids 2–167) was PCR-amplified and cloned into a pET-15b plasmid by Gibson assembly. The construct was composed of DPS fused to an N-terminal hexahistidine tag and TEV proteolytic site (Ser2 in the P7 site). The expression vector was transformed into *E. coli* BL21-CodonPlus(DE3)-RIL cells (Stratagene) and selected on ampicillin-containing LB agar. 2×YT_Amp_ medium was inoculated with a single colony and cultured overnight at 37°C with shaking. TB_Amp_ medium was then inoculated 1:50(*v*:*v*) with pre-culture and grown at 25°C. At an OD_600 nm_ of 0.8, DPS expression was induced with 0.1 m*M* IPTG for 18 h. Cells (∼20 g l^−1^) were harvested by centrifugation and resuspended 1:1(*v*:*v*) in lysis buffer (50 m*M* Tris–Cl^−^ pH 8.0 at 4°C, 500 m*M* NaCl). Cell suspensions were either processed immediately or flash-frozen in liquid nitrogen and stored at −80°C.

### 2.3. DPS purification   

**Figure 3:**
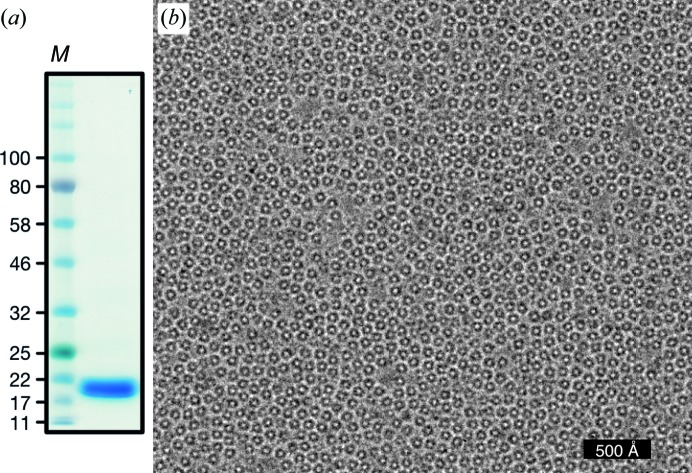
Purification of DPS from *E. coli*. (*a*) DPS is a homododecamer of 20 kDa peptides. The Coomassie-stained denaturing gel shows DPS as purified after ion-exchange chromatography. Lane *M* contains molecular-weight markers (labelled in kDa). (*b*) Representative micrograph (100 kV, cropped from full frame) of DPS particles in unsupported vitreous ice.

Cells amounting to 1 l of culture were diluted 1:4(*w*:*v*) with lysis buffer supplemented with 2.5 m*M* MgCl_2_, 20 µg ml^−1^ DNase I, 100 µg ml^−1^ lysozyme and protease-inhibitor cocktail tablets (Roche) and allowed to stir on ice for 30 min. The cells were lysed by sonication for 5 min at a 33% duty cycle (3 s on, 6 s off) and clarified by centrifugation at 50 000*g* for 20 min. The supernatant was passed through a 0.45 µm filter and supplemented with 10 m*M* imidazole before loading onto a 5 ml HisTrap FF column (GE Healthcare) at 2 ml min^−1^ at 4°C. The column was washed with 20 column volumes of lysis buffer supplemented with 40 m*M* imidazole before eluting with five column volumes of buffer containing 500 m*M* imidazole. The eluate was treated with 5 mg TEV protease (+0.5 m*M* TCEP) and transferred to 3.5 kDa molecular-weight cutoff (MWCO) Snakeskin dialysis tubing (Thermo Fisher Scientific). Imidazole was removed by dialysis against 20 m*M* Tris–Cl^−^ pH 7.5 at 20°C, 100 m*M* NaCl, 0.5 m*M* TCEP with two changes of buffer. In order to remove any residual DNA-bound DPS, the sample was passed through a 5 ml HiTrap DEAE FF column (GE Healthcare). The flowthrough was supplemented with 10 m*M* imidazole and subjected to negative chromatography on a 5 ml HisTrap FF column. The final flowthrough was concentrated to ∼1.5 ml using a 10 kDa MWCO Amicon Ultra-15 (Millipore Sigma). Approximately one third of the sample was injected at 0.5 ml min^−1^ onto a Superdex 200 Increase 10/300 GL column (GE Healthcare) equilibrated in 10 m*M* HEPES–Na^+^ pH 7.5, 100 m*M* NaCl. DPS eluted predominantly as a single major peak with a calibrated retention volume equivalent to 205 kDa or a 5.1 nm Stokes radius and with an estimated purification yield of 30–40 mg DPS per litre of culture. The purified DPS runs as a single band around 20 kDa, corresponding to the monomer of the homododecamer, on Tris-Glycine 4–20% SDS–PAGE (Fig. 3 ▸).

### 2.4. Single-particle cryoEM specimen preparation   

Human haemoglobin (Sigma, catalogue No. H7379) was resuspended in PBS (125 m*M* NaCl, 8 m*M* NaH_2_PO_4_ pH 7.4) to 7.2 mg ml^−1^ concentration. Horse (*Equus caballus*) spleen apoferritin (Sigma, catalogue No. A3660) was buffer-exchanged into PBS at pH 7.4 and adjusted to 12.1 mg ml^−1^ concentration. This specimen was only used for preliminary detector testing and was not used for high-resolution data collection. Commercially available catalase from human erythrocytes (Sigma, catalogue No. C3556) was used at 2 mg ml^−1^ concentration as supplied in 50 m*M* Tris–HCl. DPS, purified as described above, was buffer-exchanged into 20 m*M* HEPES pH 7.7, 150 m*M* KCl to a final concentration of 0.6 mg ml^−1^. Purified hepatitis B virus capsids were provided by Jan Löwe and were adjusted to 9.6 mg ml^−1^ concentration (in 50 m*M* Tris–HCl, 150 m*M* NaCl pH 7.4). *E. coli* 70S ribosomes were purified by published ultracentrifugation methods (Brown *et al.*, 2016 ▸) and were used at 3.5 mg ml^−1^ concentration (in 10 m*M* Tris pH 7.5, 50 m*M* potassium chloride, 10 m*M* ammonium chloride, 10 m*M* magnesium acetate, 6 m*M* β-mercaptoethanol). All cryo-specimens were prepared on all-gold supports (UltrAuFoil R0.6/1, 300 mesh, Quantifoil) with ∼800 nm hole diameter treated with 9:1 Ar:O_2_ plasma (in a Fischione 1070 chamber) for 60 s to render them hydrophilic. Specimens were vitrified by plunge-freezing in a 4°C cold room using a manual plunger of the Talmon type (Bellare *et al.*, 1988 ▸) and a liquid-ethane cryostat set to −181°C (Russo *et al.*, 2016 ▸). A sample volume of 3 µl was pipetted onto the foil side of the grid, the droplet was blotted for 15 s from the same side and the grid was plunged into the liquid ethane. The specimens were stored in liquid nitrogen until imaging.

### 2.5. Data collection and processing   

#### 2.5.1. Objective lens aberration measurements at 100 keV   

We measured the coefficients of spherical aberration (*C*_s_) and chromatic aberration (*C*_c_) for the Tecnai F20 electron microscope used for these experiments, equipped with a TWIN-type objective lens and operated at 100 keV. All micrographs for these measurements were recorded on an Orius phosphor-coupled CCD (Gatan).

We used a platinum/iridium specimen (Agar S114) with an ∼400 Å thick gold film evaporated on half of its grid squares. Firstly, the column was aligned to reduce the beam tilt to less than 0.5 mrad by performing sequential tableaux (Zemlin *et al.*, 1978 ▸) at around 0.5 µm defocus. The pixel size was accurately calibrated using the reflections from the gold crystals. The amount of beam tilt was calibrated by measuring the shift of the diffraction pattern of the gold when changing the rotation-centre alignments by a constant value. The spherical aberration coefficient was then measured via a Zemlin tableau with 4.8 mrad beam tilt in eight directions using the platinum/iridium specimen. A beam-tilt angle of β causes an apparent CTF overfocus of Δ*F* = 2*C*_s_β^2^ (McFarlane, 1975 ▸). The CTFs were fitted to all patterns from the tableau using *Gctf* (Zhang, 2016 ▸). The tableau was repeated three times, and the average value of the fitted defoci for the tilted-beam micrographs was subtracted from the average value of the fitted defoci for the central (near-zero beam tilt) micrograph. The principal limitation to the accuracy of this measurement is the ∼100 Å accuracy of the CTF fits and the ∼0.2 mrad accuracy of the applied beam tilt. Based on this measurement, we estimate the *C*_s_ to be 2.2 ± 0.2 mm. The fitted defocus values depend weakly on the initial *C*_s_ value used for the fit; in this case, values between 1 and 2 mm consistently yielded the same (within error) *C*_s_ estimate from the tableau. The measured value is in agreement with the value reported (*C*_s_ = 2 mm) for this microscope at 200 keV. The measured *C*_s_ value was used for CTF fitting when processing the cryoEM data.

The chromatic aberration coefficient was extracted from the dependence of the defocus on the accelerating voltage. The accelerating voltage was varied from 98 to 102 kV in 1 kV increments, and micrographs of the same platinum/iridium specimen at the same position were recorded; this was repeated three times. The CTFs were fitted using *Gctf*, and from these fits we determined that the defocus increases by 11 Å for every 1 eV increase in the accelerating voltage. This is related to the chromatic aberration *C*_c_ by
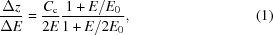
where Δ*z* is the defocus change for energy change Δ*E* around a value of *E* = 100 keV, and *E*_0_ is the rest energy of the electron (511 keV) (Reimer & Kohl, 2008 ▸). Based on this measurement, we determined a chromatic aberration *C*_c_ of 2.0 ± 0.2 mm. Although we could not measure the energy spread on the F20 used for this work directly, we estimate, based on previous measurements from a similar gun design operated under similar conditions (extraction voltage and temperature) at 100 keV on a Polara equipped with a calibrated energy spectrometer, that the source-energy spread for all of the data collected here was in the range 1.0–1.3 eV. We note that Schottky guns of this type can be operated under conditions of lower spread (Δ*E* = 0.6–0.8 eV) but we did not do this here. As is evident from the denominator of the equation, even with the same value of *C*_c_ the effect of this aberration is approximately three times stronger at 100 kV than at 300 kV.

#### 2.5.2. Data collection   

All data collection was performed on a Tecnai F20 electron microscope operated at 100 kV and fitted with an EIGER X 500K detector [Figs. 1 ▸(*a*) and 1 ▸(*b*)] using a side-entry cryoholder (Gatan 626). The DPS, haemoglobin and catalase data sets were collected at 280 000× nominal magnification, corresponding to 1.47 Å pixel size, calibrated using Au(111) reflections in the corners of the Fourier transform of a micrograph of the gold foil. The detector was operated at 9004 frames per second, and 2.05 s exposures were fractionated into 32 fractions. The fluence was set to 0.77 e^−^ Å^−2^ per fraction to give 0.006 counts per pixel per frame on the detector. This corresponded to 12.3 e^−^ Å^−2^ s^−1^ at the sample and a total flux of 24.6 e^−^ Å^−2^. The 70S ribosome and hepatitis B virus capsid data sets were collected at 150 000× nominal magnification, corresponding to a 2.74 Å pixel size, calibrated by fitting the crystal structure of the 70S ribosome to the map. To achieve optimal flux on the detector pixels, the fluence was set to 0.52 e^−^ Å^−2^ per fraction for these data sets. This corresponded to 8.3 e^−^ Å^−2^ s^−1^ at the sample and a total flux of 16.6 e^−^ Å^−2^. All data sets were collected manually using the custom interface described above; the defocus was varied between approximately 0.5 and 1.5 µm for optimal CTF fitting. All micrographs for all specimens were collected from the centres of the foil holes, where the ice is thinnest (around 300 Å or less for the smaller particles).

#### 2.5.3. Processing   

**Figure 4:**
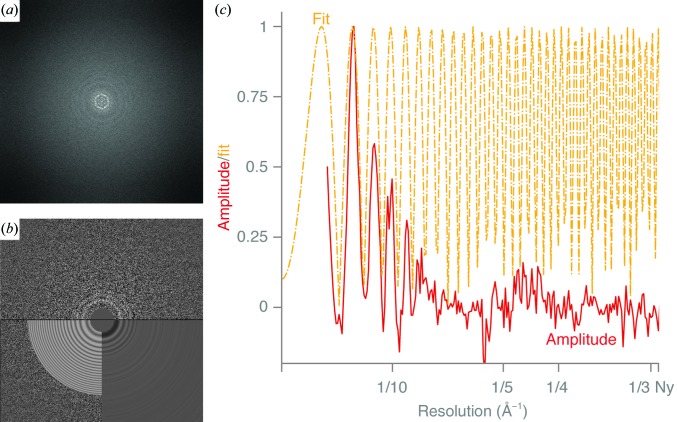
Contrast transfer function (CTF). (*a*) Fourier transform of a typical DPS micrograph. The form factor of the molecules and their close-packed ordering in the ice give the strong signals at low frequencies. (*b*) Fourier transform of the same DPS micrograph, with equiphase averaged Thon rings, and the corresponding CTF fit. (*c*) Background-subtracted amplitude spectrum (100 keV, ∼1 µm defocus, fitted in *CTFFIND*4.1, scaled to have a value of 1 at the second peak) (solid red line) and the fit (yellow dashed and dotted line).

A gain reference was produced by averaging all frames of all exposures for each data set and was applied to all movies. Defects on the detector were corrected by replacing the pixel values with the average of the adjacent pixels. All movies were motion-corrected using the *RELION*-3 implementation of motion correction (Zivanov *et al.*, 2018 ▸). The typical stage-drift rate (uniform motion in a single direction) during these exposures was found to be 7 Å s^−1^. Dose-weighting was applied, as implemented in *RELION*-3, by scaling the radiation-damage rate assumed at 300 kV by a factor of 1.57, as measured for the inelastic electron scattering cross-section from carbon at 100 kV compared with 300 kV (Peet *et al.*, 2019 ▸). Power spectra were calculated for groups of every three frames. CTFs were fitted to these power spectra in the 25–4 Å resolution range using *CTFFIND* 4.1 (Rohou & Grigorieff, 2015 ▸) with amplitude contrast 0.07 (Toyoshima & Unwin, 1988 ▸) and spherical aberration 2.0 mm (Fig. 4 ▸). Particles were manually picked in *EMAN*2 *boxer* (Tang *et al.*, 2007 ▸) and all further processing was performed in *RELION*-3.

**Figure 5:**
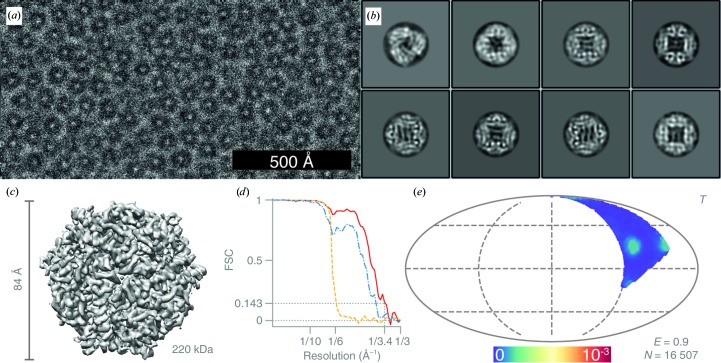
Structure of DPS determined at 100 keV. (*a*) Typical micrograph of DPS after motion correction. Contrast is adjusted to ±3σ from the mean intensity. (*b*) 2D class averages. (*c*) Sharpened masked 3D reconstruction of DPS from *N* = 16 507 particles with tetrahedral (*T*) symmetry. (*d*) Gold-standard FSC plot corresponding to the calculated map, showing the correlation between the phase-randomized (yellow), unmasked (blue) and masked (red) half-maps. The plot terminates at the Nyquist frequency. (*e*) Orientation distribution of the DPS particles contributing to the final reconstruction (Mollweide projection). All Euler angles are assigned within one asymmetric subunit for the tetrahedrally (*T*) symmetric particle. The colour scale (linear) corresponds to the normalized density of views (blue, low; red, high). *E* = 0.9 is the efficiency of the orientation distribution, indicating nearly uniform sampling of the particle views.

For the DPS data set, 68 817 particles were manually picked from 739 micrographs [Fig. 5 ▸(*a*)], extracted in 128 × 128 pixel boxes and subjected to sequential rounds of 2D classification with *T* = 1 and with CTFs ignored to the first peak. Approximately half of the particles were discarded during classification; representative classes that were retained are shown in Fig. 5 ▸(*b*). The rest of the particles were subjected to initial 3D refinement with CTFs ignored to the first peak. The initial model for the refinement was generated from the crystal structure (PDB entry 1dps; Grant *et al.*, 1998 ▸), low-pass filtered to 20 Å, with tetrahedral (*T*) symmetry imposed. After 3D classification, 16 507 particles were selected for the next round of 3D refinement. The resulting 4.8 Å resolution map was used for per-micrograph defocus and astigmatism refinement, and estimation of beam tilt and higher order aberrations. These parameters were calculated separately for data acquired on different days. After particle polishing, the final 3D map was calculated. The resulting map was sharpened with a *B* factor of −132 Å^2^, as fitted to the Guinier plot (Rosenthal & Henderson, 2003 ▸) [Fig. 5 ▸(*c*)].

**Figure 6:**
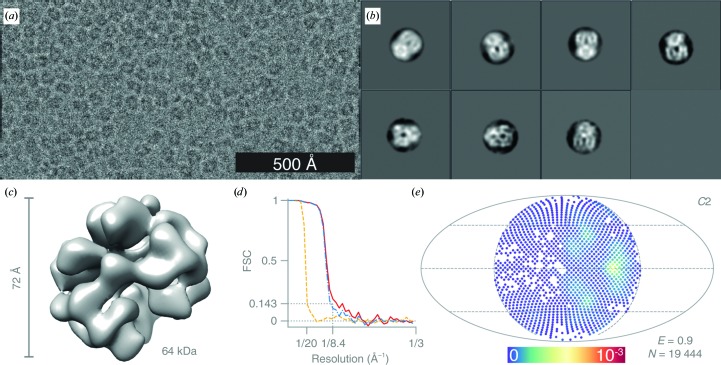
Structure of haemoglobin determined at 100 keV. (*a*) Typical micrograph after motion correction. Contrast is adjusted to ±3σ from the mean intensity. (*b*) 2D class averages. (*c*) Sharpened masked 3D reconstruction of haemoglobin from *N* = 19 444 particles with *C*2 symmetry. (*d*) Gold-standard FSC plot corresponding to the calculated map, showing the correlation between the phase-randomized (yellow), unmasked (blue) and masked (red) half-maps. The plot terminates at the Nyquist frequency. (*e*) Orientation distribution of the haemoglobin particles contributing to the final reconstruction (Mollweide projection). All Euler angles are assigned within one asymmetric subunit for the *C*2 symmetric particle. The colour scale (linear) corresponds to the normalized density of views (blue, low; red, high). *E* = 0.9 is the efficiency of the orientation distribution, indicating nearly uniform sampling of the particle views.

For the haemoglobin data set, 64 986 particles were manually picked from 576 micrographs [Fig. 6 ▸(*a*)], extracted in 80 × 80 pixel boxes and subjected to sequential rounds of 2D classification with *T* varying between 1 and 1.5 and with CTFs ignored to the first peak. The subset of 19 444 particles that produced 2D classes with secondary-structure features [Fig. 6 ▸(*b*)] were subjected to 3D refinement, with CTFs ignored to the first peak, against the low-pass filtered (to 15 Å) EMD-3488 map (Khoshouei *et al.*, 2017 ▸) with *C*2 symmetry imposed. The resulting map was manually sharpened with a *B* factor of −300 Å^2^ [Fig. 6 ▸(*c*)].

**Figure 7:**
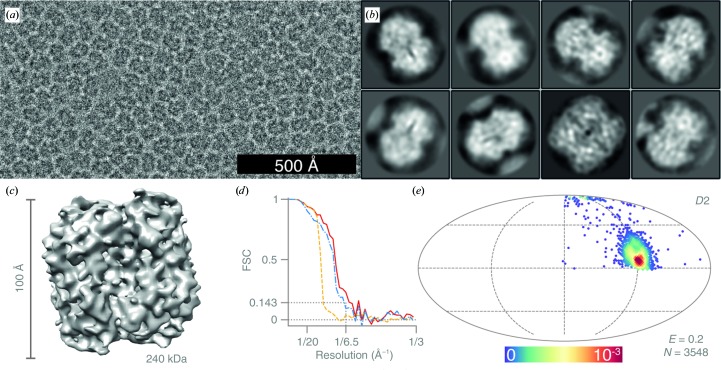
Structure of catalase determined at 100 keV. (*a*) Typical micrograph after motion correction. Contrast is adjusted to ±3σ from the mean intensity. (*b*) 2D class averages, showing mostly side views, except for a single class showing the strongly under-represented top view. (*c*) Sharpened masked 3D reconstruction of catalase from *N* = 3548 particles with dihedral (*D*2) symmetry. (*d*) Gold-standard FSC plot corresponding to the calculated map, showing the correlation between the phase-randomized (yellow), unmasked (blue) and masked (red) half-maps. The plot terminates at the Nyquist frequency. (*e*) Orientation distribution of the catalase particles contributing to the final reconstruction (Mollweide projection). All Euler angles are assigned within one asymmetric subunit for the *D*2 symmetric particle. The colour scale (linear) corresponds to the normalized density of views (blue, low; red, high). *E* = 0.2 is the efficiency of the orientation distribution, indicating a strong orientation preference, which limits the resolution of the reconstruction.

For the catalase data set, 11 948 particles were manually picked from 133 micrographs [Fig. 7 ▸(*a*)] and extracted in 80 × 80 pixel boxes. The particles were subjected to one round of 2D classification with *T* = 1 [Fig. 7 ▸(*b*)], followed by 3D classification with *T* = 3, after which the final 3548 particles for 3D refinement were selected. The 3D refinement was performed against a previously calculated low-resolution EM density map (unpublished work) filtered to 15 Å and *D*2 symmetry was applied. The map was manually sharpened with a *B* factor of −450 Å^2^ [Fig. 7 ▸(*c*)].

**Figure 8:**
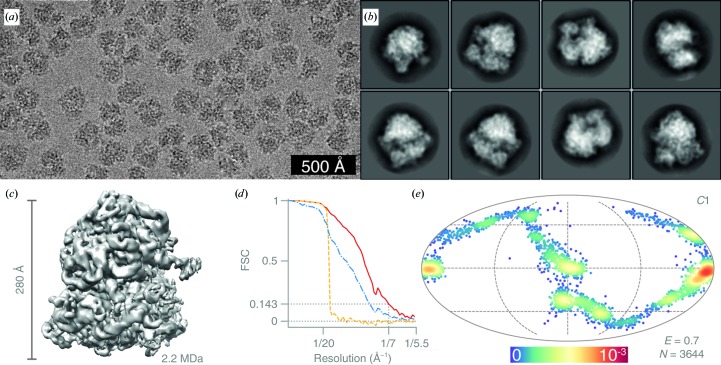
Structure of the *E. coli* 70S ribosome determined at 100 keV. (*a*) Typical micrograph after motion correction. Contrast is adjusted to ±3σ from the mean intensity. (*b*) 2D class averages. (*c*) Sharpened masked 3D reconstruction of the 70S ribosome from *N* = 3644 particles without symmetry (*C*1). (*d*) Gold-standard FSC plot corresponding to the calculated map, showing the correlation between the phase-randomized (yellow), unmasked (blue) and masked (red) half-maps. The plot terminates at the Nyquist frequency. (*e*) Orientation distribution of the 70S ribosomes contributing to the final reconstruction (Mollweide projection). The colour scale (linear) corresponds to the normalized density of views (blue, low; red, high). *E* = 0.7 is the efficiency of the orientation distribution.

For the 70S ribosome data set, 7560 particles were manually picked from 127 micrographs [Fig. 8 ▸(*a*)] and extracted in 164 × 164 pixel boxes. The particles were subjected to 2D classification with *T* = 2 and the classes showing secondary structure [Fig. 8 ▸(*b*)] were selected for initial 3D refinement against a published EM map (EMD-4476; Rae *et al.*, 2019 ▸), followed by 3D classification. After that, a subset of 3644 particles was selected for final 3D refinement. The map was manually sharpened with a *B* factor of −150 Å^2^ [Fig. 8 ▸(*c*)].

**Figure 9:**
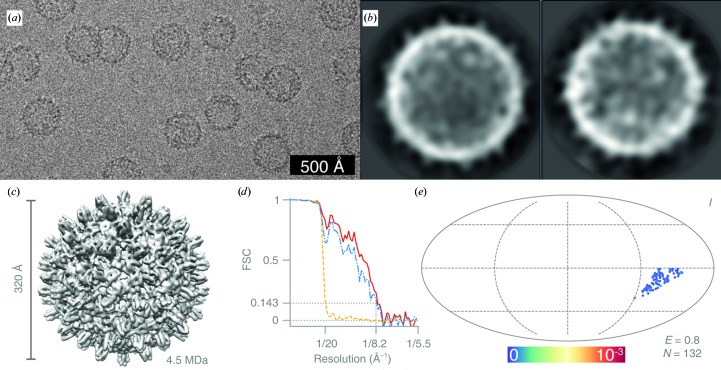
Structure of hepatitis B virus capsid determined at 100 keV. (*a*) Typical micrograph after motion correction. Contrast is adjusted to ±3σ from the mean intensity. (*b*) 2D class averages. (*c*) Sharpened masked 3D reconstruction of hepatitis B virus capsid from *N* = 132 particles with icosahedral (*I*) symmetry. (*d*) Gold-standard FSC plot corresponding to the calculated map, showing the correlation between the phase-randomized (yellow), unmasked (blue) and masked (red) half-maps. The plot terminates at the Nyquist frequency. (*e*) Orientation distribution of the hepatitis B capsids contributing to the final reconstruction (Mollweide projection). All Euler angles are assigned within one asymmetric subunit for the icosahedrally (*I*) symmetric particle. The colour scale (linear) corresponds to the normalized density of views (blue, low; red, high). *E* = 0.8 is the efficiency of the orientation distribution, indicating nearly uniform sampling of the particle views.

For the hepatitis B virus capsid data set, 167 particles were manually picked from 23 micrographs [Fig. 9 ▸(*a*)] and extracted in 140 × 140 pixel boxes. The particles were subjected to one round of 2D classification with *T* = 1 and with the CTF ignored to the first peak. The 132 particles from the selected 2D classes [Fig. 9 ▸(*b*)] were refined in 3D against a previously determined EM map (unpublished) filtered to 30 Å with icosahedral symmetry imposed. The resulting map was manually sharpened with a *B* factor of *B* = −300 Å^2^ [Fig. 9 ▸(*c*)].

The efficiency of all resulting orientation distributions was estimated using *cryoEF* (Naydenova & Russo, 2017 ▸). All orientation distributions in Figs. 5 ▸(*e*), 6 ▸(*e*), 7 ▸(*e*), 8 ▸(*e*) and 9 ▸(*e*) are shown on the same colour scale, representing the normalized probability density of each observed orientation.

## 3. Results   

We determined the structures of five biological specimens, hepatitis B virus capsid, *E. coli* 70S ribosome, catalase, haemoglobin and DPS, in seven days of data collection at 100 kV using the EIGER X 500K detector. Replacing the detector on the microscope took one day, as did reverting the microscope to its original configuration at the end of these experiments. During the data collection, 97% of the pixels on the detector were usable. A four-pixel-wide rim around the edge of the detector and an 8 × 256 pixel stripe across the field of view were not usable, with constant high-number readout. In addition to the defective stripes, four pixels at each of the eight locations of the ASIC boundaries around the edge had a fixed output. All these defective pixels were replaced with the average value of the neighbouring pixels after gain correction.

The highest resolution structure that we determined is that of the 220 kDa protein DPS with tetrahedral symmetry, which reached 3.4 Å resolution from 16 500 particles. At this resolution, the density for most side chains is sufficiently clear to allow model building. From the 2D class averages, the twofold and threefold symmetric views can be distinguished. To obtain 2D classes with clear secondary-structure features, we had to ignore the CTF values up to the first peak owing to the strong form-factor signal of the particles [Fig. 4 ▸(*a*)]. Despite the close packing of the particles in the amorphous ice, they could still be aligned to yield the final reconstruction. We note the poor accuracy of the initial CTF fits, compromised by the small field of view and the small number of pixels on the detector used in these experiments. The CTF fits were improved using per-micrograph defocus and astigmatism refinement in *RELION*-3 (Zivanov *et al.*, 2018 ▸). The resolution of the final map was improved from 4.8 to 3.4 Å by correcting for the beam tilt, which varied between 0.5 and 3.0 mrad, and higher order aberrations on each group of micrographs acquired on different days. The final DPS map and the full raw data set have been deposited in the EMDB (EMD-10161) and EMPIAR (EMPIAR-10297), and the measured MTF of the detector is provided as supporting information to aid other researchers who wish to use these data for the development of better algorithms and software for 100 keV cryoEM.

The structure of catalase from human erythrocytes (240 kDa, *D*2 symmetry) was determined nominally to 6.5 Å resolution, calculated by the gold-standard FSC at 0.143 (Harauz & van Heel, 1986 ▸; Rosenthal & Henderson, 2003 ▸; Scheres & Chen, 2012 ▸). The actual resolution of the map is strongly anisotropic, ranging from better than 4 Å in the plane of the preferred view to 10 Å in the orthogonal direction, owing to the preferred orientation exhibited by this protein, *i.e.* the low efficiency (*E* = 0.2) of the orientation distribution [Fig. 7 ▸(*e*)].

The larger specimens ribosomes (Fig. 8 ▸) and hepatitis B virus capsid (Fig. 9 ▸) were imaged at a lower magnification to accommodate a larger number of particles per micrograph and represent the typical screening approach prior to high-resolution data collection. The Nyquist frequency for these data sets was 5.48 Å. We obtained 2D class averages showing secondary-structure features, and in the test case of the 70S ribosome we could separate out classes for the 50S subunits alone as well. The three-dimensional reconstructions reached 8.2 Å from 132 hepatitis B virus capsid particles and 7.0 Å from 3644 ribosomes. The final ribosome map and the full raw data set have also been deposited in the EMDB (EMD-10265) and EMPIAR (EMPIAR-10316). Blurring of the 30S ribosomal subunit owing to the conformational heterogeneity of the sample can be seen in the 2D class averages and in the final map. The orientation distribution of the 70S ribosomes clearly shows the particles adopting distinct preferred orientations. Data collection in this configuration can be used to optimize the specimen of interest on different supports. The fidelity of the particles, the presence or absence of particular sub­complexes or molecules and the quality of the orientation distribution (efficiency) can all be easily determined from data sets such as these.

We demonstrate that we can also image specimens at the lowest size limit of cryoEM. We determined the structure of haemoglobin (64 kDa, with *C*2 symmetry) to 8.4 Å resolution, where clear α-helix separation can be observed. At this resolution one could distinguish, for example, the conformational difference between oxyhaemoglobin and deoxyhaemoglobin.

The microscope, as configured for this study, is capable of resolving a 3.4 Å lattice in all directions at 100 keV on a standard test specimen such as graphitized carbon without the use of tilt and the 2.35 Å gold lattice. Since the spherical aberration of the lens is corrected for accurately during the reconstruction process, we do not expect it to pose any limitations to high-resolution imaging of biological specimens at 100 keV. In contrast, the effect of chromatic aberration increases at lower accelerating voltages owing to the increase in the fractional energy spread Δ*E*/*E* and the increase in the electron wavelength λ. Comparing electron beams with the same energy spread Δ*E*, the envelope function *K*_c_(*q*) that damps the CTF oscillations at spatial frequency *q* is
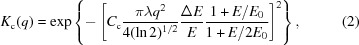
which decays to 1/*e* at an approximately 2.2× lower spatial frequency at 100 kV than at 300 kV (Reimer & Kohl, 2008 ▸). Similarly, the phase errors owing to beam tilt become more pronounced at 100 keV than at 300 keV owing to the longer wavelength of the electrons; these can be corrected for in the software as well, as shown in the example of the DPS data set.

## 4. Discussion   

We have demonstrated that a 100 keV electron microscope equipped with a field-emission gun (FEG) and a direct electron detector is suitable for imaging vitrified biological specimens at high resolution. Such an instrument is especially useful for screening specimens for suitable ice thickness, protein quality and orientation distribution efficiency. All the reconstructions reported here achieved sufficient resolution to provide useful information about the specimen orientation in the micrographs; the same strategy of quick reconstructions from small data sets can be used, for example, to optimize the choice of specimen supports and grid-freezing conditions for high-resolution data collection. Specimen evaluation and optimization could be greatly accelerated if inexpensive 100 kV instruments with an FEG source and a direct detector were readily available. With the example of DPS, we also demonstrate that a 3.4 Å resolution structure can be determined using such an instrument.

The current technical limitation to high-resolution imaging at 100 keV is the small detector area. The micrographs shown in Figs. 5 ▸(*a*), 6 ▸(*a*) and 7 ▸(*a*) are from 150 × 75 nm areas on the specimen; this is 32 times smaller than the area that could be recorded on a 4k × 4k detector at the same magnification. All of the specimens used in these experiments were prepared at near the maximal possible concentration while still separating discrete particles in the vitreous ice; even so, the power in the amplitude spectrum of the micrographs was suboptimal for accurate CTF fitting. The development and commercialization of larger area, fast direct electron detectors optimized for 100 keV should soon enable microscopists to use the improvement in signal-to-noise (SNR) ratio per unit damage offered by the lower energy electrons. We emphasise the importance of specimen preparation, including minimizing the ice thickness, for maximal improvement of the SNR. We have deposited the full 70S ribosome and DPS data sets (raw movie stacks) in the EMPIAR database to facilitate software optimization for 100 keV data processing. For example, the lower accelerating voltage corresponds to a higher Ewald sphere curvature; at the resolutions considered here this effect is negligible, but at higher resolutions it can be fully corrected after acquisition (Russo & Henderson, 2018*a* ▸), as implemented in *RELION*-3 (Zivanov *et al.*, 2018 ▸).

### 4.1. Affordable cryoEM   

We have shown in this demonstration that it is possible to determine subnanometre structures of a variety of vitrified biological specimens by cryoEM using 100 keV electrons. The experiments have also sharpened our appreciation of the key factors needed to produce an affordable electron cryomicroscope, with the aim of greatly broadening the accessibility of cryoEM to the structural biology community worldwide in both academia and industry. The following is a list of the key requirements for such an instrument.

(i) High vacuum (<10^−7^ Torr) with a good anti-ice cryo-shield surrounding the specimen (Homo *et al.*, 1984 ▸). It is easy to show that at 10^−6^ Torr there would be 10^15^ water molecules per cm^2^ (a monolayer) per second striking all surfaces in the vacuum, and a tenth of a monolayer per second at 10^−7^ Torr. An ice-contamination rate of less than 7 Å per hour would allow the careful examination of single cryoEM grids while retaining the option of continuing data collection for a period of up to 8 h. A cryostage with a reduced drift rate (so that the total drift is <1 Å during a typical exposure) would facilitate high-resolution data collection.
(ii) It is essential to have an FEG source (Schottky or cold) to provide sufficient spatial coherence to take 1–2 s exposures that allow the use of enough defocus to give low-resolution contrast while retaining high-resolution detail (Russo & Henderson, 2018*b* ▸; Börrnert *et al.*, 2018 ▸).
(iii) A small chromatic aberration coefficient, *C*_c_, for the objective lens is a more stringent requirement at 100 keV than at 300 keV (equations 1 and 2), if the resolution requirement of the microscope needs to include the ability to reach a resolution beyond 3 Å, which may be useful for some projects. For a source with energy spread Δ*E* ≲ 1 eV, a *C*_c_ of less than 2 mm would be adequate, which is available on existing microscopes. This places a restriction on the pole gap and bore, but can easily be accommodated provided that large specimen tilts are not required. A tilt range of ±10° would be useful for single-particle cryoEM, but electron tomography would not be possible with 3 mm foils.
(iv) As shown here, the most urgent need is for the development of a large-area direct electron detector that is optimized to give a high DQE at 100 keV. At present, phosphor/fibre-optics cameras work reasonably well but have DQEs that are too low for effective cryoEM. The Dectris EIGER X 500K detector used in this paper has excellent performance, but the active area is too small for convenient and efficient use. A purpose-designed detector (CMOS or hybrid pixel) with a square area of 2000 or 3000 pixels on edge would be ideal, 8× to 16× larger than the detector used in this paper. The main constraint for recording high-DQE images at 100 keV arises from the greatly increased electron scatter in the detector, for which the simplest solution is to develop a design that uses a larger pixel whose size matches the range of electron scattering in the detector.
(v) To reach a DQE(0) that is near 100%, every electron that deposits energy in the detector should be counted individually (Turchetta, 1993 ▸; Li *et al.*, 2013 ▸; McMullan *et al.*, 2014 ▸). This was not performed in the present work, but with improvements to the data-acquisition system (DAQ) the current detector would have an almost perfect DQE: better than 0.97 at zero spatial frequency and better than 0.5 at the Nyquist frequency. The same constraints apply to the DAQs for both monolithic active pixel sensors using CMOS technology and to hybrid pixel sensors such as the EIGER used here.
(vi) For wide adoption, an affordable electron cryomicroscope should be easy to use and have modest environmental requirements from the laboratory in which it is housed. This includes relative insensitivity to electric and magnetic fields, vibrations and temperature fluctuations. The physical size, installation and maintenance requirements should also be moderate, such that most laboratory spaces would be suitable. Reducing the accelerating voltage to 100 kV and targeting a resolution of 3 Å makes this easier to accomplish.

### 4.2. Ultimate cryoEM   

Finally, one can consider what would be required to create a 100 keV electron microscope that could routinely achieve better than 2 Å resolution on biological specimens. Previous work on radiation-hard specimens such as graphene have shown that <1 Å resolution is possible at 80 keV (Bell *et al.*, 2010 ▸), 40 keV (Bell *et al.*, 2012 ▸) and even 20 keV (Linck *et al.*, 2016 ▸; Börrnert & Kaiser, 2018 ▸). It is clear from this work and from other recent results using post-data-collection aberration correction in software (Zivanov *et al.*, 2018 ▸) that the limiting factor is the chromatic aberration of the lens. There are several ways to mitigate the effects of *C*_c_, as is evident from (1); we consider them in order of increasing cost and complexity. The first and simplest is to reduce the extraction voltage and temperature of a Schottky-type FEG to their minimum, thus reducing the energy spread to around 0.7–0.5 eV. Secondly, one can use a cold FEG, which reduces the energy spread of the source even further but requires better vacuum and periodic flashing to refresh the integrity of the tip. Thirdly, one can employ the use of a monochromator to reduce the energy spread of either type of source to <100 meV. This has been shown to increase the bounds of the envelope function and improve the resolution of a Schottky-type source to 1 Å (Bell *et al.*, 2010 ▸; Mukai *et al.*, 2014 ▸). Fourthly, an objective lens design can be optimized for minimal *C*_c_ at the expense of other parameters such as the gap distance. Finally, a full hardware *C*_c_ corrector can be added to reduce the *C*_c_ of the image-forming lens to an arbitrarily low value and thus eliminate this part of the envelope function entirely (Rose, 2009 ▸; Zach, 2009 ▸; Kabius *et al.*, 2009 ▸; Haider *et al.*, 2010 ▸). We envision that future cryomicroscopes designed for ultimate resolution in single-particle work will be designed both to maximize the information coefficient (information available per unit damage; Peet *et al.*, 2019 ▸) and minimize the presence or effects of *C*_c_ along with other geometric aberrations of the objective lens as necessary. In some instruments this may involve the use of a monochromator (Krivanek *et al.*, 2009 ▸; Essers *et al.*, 2010 ▸; Tsuno, 2011 ▸) or a cold FEG (Crewe *et al.*, 1968 ▸) to reduce the energy spread of the source with a small gap lens, or in the case of high-end instruments a *C*_c_ corrector to reduce the *C*_c_ to negligible values (Forbes *et al.*, 2014 ▸; Linck *et al.*, 2016 ▸). This will then allow true atomic resolution (∼1 Å) cryoEM on appropriate specimens where the additional cost and effort required to achieve these resolutions is deemed appropriate and necessary to solve the biological problem of interest.

## 5. Conclusions   

The subnanometre single-particle cryoEM structures described in this paper provide a practical demonstration that high-quality cryoEM can be performed at 100 keV with relatively unsophisticated equipment. Currently, the lack of a suitable high-speed, high-efficiency detector with a large number of pixels ( >4 × 10^6^) is the primary limitation both to the ultimate achievable resolution and to the practical use of a 100 keV transmission electron microscope for cryoEM. With additional investment in the development of improved 100 keV detectors and field-emission sources, the use of cryoEM in structural biology could be greatly expanded. Further, a path to creating a fully optimized, atomic resolution electron cryomicroscope, which can rapidly determine the structures of single-particle specimens, is now clear.

## Supplementary Material

EMDB reference: DNA protection during starvation protein, EMD-10161

EMDB reference: bacterial 70S ribosome, EMD-10265

EIGER X 500K detector 100 keV MTF. DOI: 10.1107/S2052252519012612/eh5004sup1.txt
